# Perceptions, Practices, and Challenges of Interceptive Orthodontics Among Indian Pediatric Dentists: A Cross-Sectional Survey

**DOI:** 10.7759/cureus.98342

**Published:** 2025-12-02

**Authors:** KC Vignesh, Vivien Ramsey, Athiya Fathima, Selvakumar Haridoss, Rajkumar Manoharan, Priya J, Kavitha Swaminathan

**Affiliations:** 1 Pediatric and Preventive Dentistry, Sri Ramachandra Institute of Higher Education and Research, Chennai, IND; 2 Pedodontics and Preventive Dentistry, Sri Ramachandra Institute of Higher Education and Research, Chennai, IND; 3 Public Health Dentistry, Sri Ramachandra Institute of Higher Education and Research, Chennai, IND

**Keywords:** #cross-sectional survey, early orthodontic treatment, interceptive orthodontics, pediatric dentistry, perceptions and barriers, sdg 10: reduced inequalities, sdg 3: good health and well-being, sdg 4: quality education

## Abstract

Background

Interceptive orthodontics in the primary and mixed dentitions can reduce the severity of developing malocclusions and simplify later comprehensive treatment. Pediatric dentists are ideally placed to recognize early malocclusion and initiate or coordinate interceptive care, yet data on how they perceive and implement interceptive orthodontics in India are limited.

Aim

This study aimed to evaluate Indian pediatric dentists’ perceptions, clinical practices, and perceived barriers related to interceptive orthodontics.

Materials and methods

A descriptive, questionnaire-based, cross-sectional survey was conducted among pediatric dentists across India (July-October 2025). A self-administered, validated online questionnaire assessed sociodemographic details, awareness, and perceptions of interceptive orthodontics, clinical practice patterns, and perceived barriers and training needs. The survey link was circulated via professional networks, alumni mailing lists, and social media platforms. Data from 204 (100.0%) complete responses were analyzed using descriptive statistics and chi-square tests to explore associations between respondent characteristics and routine provision of interceptive orthodontics.

Results

Most respondents were affiliated with academic institutions (159 (77.9%)) and routinely provided interceptive orthodontic treatment (159 (77.9%)). Habit-breaking appliances (159 (78.0%)) and space maintainers (148 (72.5%)) were the most commonly used modalities, mainly for habit-related problems, space maintenance, crossbites, and early crowding. Exactly 102 (50.0%) respondents strongly agreed, and 79 (38.7%) agreed that interceptive treatment reduces the complexity of subsequent orthodontic care; 102 (50.0%) perceived it as cost-effective, while 90 (44.2%) felt cost-effectiveness was case dependent. Key barriers (multiple responses permitted) included lack of parental understanding (128 (62.7%)), compliance issues (118 (57.8%)), difficulty managing child cooperation (116 (56.9%)), and treatment cost (91 (44.6%)). Most pediatric dentists reported being very or moderately satisfied with interceptive outcomes (102 (50.0%) and 90 (44.2%), respectively), and 136 (66.7%) were likely to continue offering these services as a first-line option. Younger age and greater clinical experience were significantly associated with a higher likelihood of routinely providing interceptive orthodontics, with non-significant trends towards higher provision among pediatric dentists working in academic settings and among female practitioners.

Conclusion

Indian pediatric dentists recognize the importance of interceptive orthodontics and already provide a core set of early orthodontic services. However, gaps in parental awareness, child compliance, and structured training may limit the breadth and consistency of care. Strengthening postgraduate curricula, continuing-education opportunities, and system-level support could help translate positive attitudes into more comprehensive and equitable interceptive orthodontic care for children.

## Introduction

Malocclusion is one of the most common oral health problems in children and adolescents, second only to dental caries, and has been linked with caries risk, periodontal problems, temporomandibular disorders, and adverse psychosocial consequences [1]. Early orthodontic care in the primary and mixed dentitions encompasses both preventive and interceptive approaches: preventive orthodontics aims to preserve normal occlusal development, while interceptive orthodontics seeks to recognize and eliminate developing irregularities such as space loss, deleterious oral habits, transverse discrepancies, and emerging skeletal disharmonies before they become established [2]. By intervening during periods of active craniofacial growth, interceptive therapy can reduce the severity of malocclusion, simplify or shorten subsequent comprehensive treatment, decrease the need for extractions or orthognathic surgery, and improve children’s self-esteem and oral health-related quality of life [2]. Pediatric dentists, who provide ongoing care from early childhood, are uniquely positioned to identify these developing problems and either institute interceptive orthodontic treatment themselves or facilitate timely referral.

Several questionnaire-based surveys have explored knowledge, attitudes, and practices related to early orthodontic intervention among different stakeholders. Acharya et al. reported that general dentists and non-orthodontic specialists in Nepal had broadly acceptable orthodontic knowledge but notable gaps in counseling and referral decisions for growing patients [1]. Hudson et al. found that South African general dental practitioners attained good knowledge scores for interceptive orthodontics but often did not provide interceptive treatment due to limited interest, perceived lack of clinical skills, infrastructure constraints, and reimbursement issues [3]. Barzilay et al. showed that pediatric specialists demonstrated significantly higher orthodontic knowledge and more appropriate referral patterns for early treatment than general dental practitioners, yet both groups were uncertain regarding the timing of intervention for maxillary canine impaction and indications for leeway space maintenance [4]. From the caregivers’ perspective, Alharbi et al. reported that only a small proportion of parents had good knowledge of interceptive orthodontic treatment despite generally positive attitudes towards early correction [5]. Importantly, Nagrale et al. evaluated the knowledge, attitude, and awareness of private practitioners in Pune regarding interceptive orthodontic intervention and reported only average levels of knowledge and practice, highlighting uncertainty about appliances, diagnostic tools, and cost-related issues in everyday practice [6].

Beyond conventional fixed and removable appliances, emerging modalities, such as clear aligners, are increasingly used in early orthodontic contexts; in our previous survey of Indian pediatric dentists, clear aligner therapy in mixed dentition was perceived as moderately effective, with aesthetics and hygiene as key advantages but cost, compliance, and eruption management identified as important barriers to wider adoption [7].

Despite these contributions, there remains a relative paucity of data specifically examining how pediatric dentists themselves perceive and integrate interceptive orthodontics into routine clinical practice, particularly in settings where access to specialist orthodontic services may be limited. Most existing surveys have focused on general practitioners, mixed groups of non-orthodontic specialists, or parents, and have seldom detailed the full spectrum of interceptive procedures, indications, and perceived challenges within pediatric dental practice [1,3-6]. Moreover, while our earlier work characterized pediatric dentists’ perceptions and practices regarding clear aligner therapy in mixed dentition [7], there is limited evidence on their broader interceptive orthodontic armamentarium, including space maintenance, guidance of eruption, transverse expansion, habit-breaking appliances, functional appliances, and serial extraction, and how these are actually deployed in daily practice. Considering the potential of interceptive orthodontics to reduce treatment complexity and burden in later years, it is necessary to understand pediatric dentists’ perceptions, current practice patterns, perceived effectiveness, barriers, and future intent regarding interceptive orthodontic care. The present study was therefore designed to address this gap by systematically evaluating pediatric dentists’ perceptions and practices related to interceptive orthodontics using a validated questionnaire to capture key domains of clinical decision-making and implementation.

## Materials and methods

Study design and ethical approval

This was a descriptive, questionnaire-based, cross-sectional study conducted among pediatric dentists across India (Appendices). The survey targeted pediatric dentists working in diverse practice settings, including academic institutions, private clinics, and government hospitals, to capture a broad national perspective on interceptive orthodontic care. Ethical approval for the study was obtained from the Ethics Committee for Student Projects, Sri Ramachandra Institute of Higher Education and Research, Chennai, India (CSP-III/25/SEP/26/398). The study adhered to the ethical principles outlined in the Declaration of Helsinki. Participation was voluntary, and informed consent was obtained electronically at the beginning of the questionnaire. The study was designed, conducted, and reported in accordance with the STROBE (Strengthening the Reporting of Observational Studies in Epidemiology) guidelines for cross-sectional studies.

Study duration and participants

Data were collected over approximately three months, from July 2025 to October 2025. Practicing pediatric dentists from various clinical and academic settings across India were invited to participate. Eligible participants were pediatric dentists currently engaged in full-time or part-time clinical practice in India, willing to provide informed consent and complete the questionnaire. Respondents were excluded if they were retired, were general practitioners or non-pediatric dental specialists, or submitted incomplete or duplicate responses.

Sample size determination and justification

The required sample size was calculated using nMaster software (version 2, Christian Medical College, Vellore, India). Assuming a 95% confidence level (α = 0.05), 80% power (β = 0.20), a precision of 6%, and a conservative anticipated response distribution of 50%, the minimum sample size was estimated to be 267 pediatric dentists.

Over the July-October 2025 data collection period, we achieved 204 complete responses, representing 76.4% of the planned sample size. Although the achieved sample size was lower than the initial target, it is comparable to, and in many cases exceeds, sample sizes used in similar survey-based studies in Indian dental literature [8,9]. The achieved sample yields an approximate margin of error of ±6.8% at the 95% confidence level, which is acceptable for exploratory questionnaire-based research. Furthermore, respondents represented a wide spread of age groups, gender, years of experience, and practice settings, with a high proportion affiliated with academic institutions, reflecting the intended focus on pediatric dental education and training networks.

Survey instrument, validity, reliability, and pilot testing

A structured, self-administered questionnaire was developed to assess pediatric dentists’ perceptions and clinical practices related to interceptive orthodontics. The instrument was designed following a comprehensive literature review on preventive and interceptive orthodontic care, early orthodontic assessment, and previous surveys among general dentists, specialists, and parents, as well as our earlier validated questionnaire on clear aligner therapy in mixed dentition.

The final questionnaire comprised sections covering sociodemographic and professional details; awareness and perceptions regarding the role, timing, and objectives of interceptive orthodontic treatment; clinical practices related to diagnosis, commonly performed procedures, and referral patterns; and perceived barriers and training needs. Items were primarily closed-ended questions (multiple-choice, yes/no, and ordinal options), enabling quantitative analysis of practice patterns and perceptions.

Face and content validity were established through review by a panel of three pediatric dentistry faculty members with expertise in interceptive orthodontics, who independently assessed each item for relevance, clarity, and coverage of the intended content domains (awareness and perceptions, diagnostic practices, clinical procedures, referral and collaboration, and perceived barriers and training needs). Their feedback was discussed and incorporated by consensus, with minor modifications to wording, sequencing, and response options.

The draft questionnaire was then pilot tested among a convenience sample of pediatric dentists who were not included in the final analysis. The pilot focused on item clarity, layout, ease of completion, and approximate completion time. Based on this feedback, small refinements were made to the wording and ordering of selected items; no major content changes were required.

Questionnaire reliability was evaluated in the pilot dataset by estimating internal consistency for a three-item perceptions/satisfaction subscale (“importance of interceptive orthodontics,” “satisfaction with outcomes,” and “likelihood of continuing to offer interceptive orthodontics as a first-line option”). Cronbach’s alpha for this subscale was 0.77, indicating acceptable internal consistency for exploratory survey work.

Data collection procedure

The finalized questionnaire was converted into an online format using Google Forms (Google LLC, Mountain View, CA), and the survey link was circulated to approximately 600 pediatric dentists across India through professional and alumni mailing lists and pediatric dentistry-focused social media/WhatsApp (Meta Platforms, Inc., Menlo Park, CA) groups. A combination of direct invitations and snowball sampling was employed, with participants encouraged to share the survey link with eligible colleagues. The form remained open throughout the July-October 2025 period, and periodic reminders were sent to enhance participation. Each respondent could submit the form only once; duplicate entries were minimized by restricting multiple submissions from the same email address and cross-checking suspiciously similar responses. No personally identifiable clinical data were collected.

Statistical analysis

Data were exported from Google Forms into Microsoft Excel (Microsoft® Corp., Redmond, WA) and then analyzed using IBM SPSS Statistics for Windows, version 26 (IBM Corp., Armonk, NY). Descriptive statistics (frequencies and percentages) were used to summarize categorical variables related to demographics, perceptions, practices, and perceived barriers. Inferential statistics, primarily chi-square tests, were employed to explore associations between key variables (e.g., years of clinical experience, practice setting, and the likelihood of performing specific interceptive procedures or referring to an orthodontist). Categories were collapsed when necessary to ensure adequate cell counts for analysis. Because the dataset consisted almost entirely of categorical variables and no continuous scale scores were generated, only non-parametric tests for categorical data were applied. A p-value < 0.05 was considered statistically significant.

## Results

Participant characteristics

A total of 204 (100.0%) pediatric dentists completed the survey (Table 1). Most respondents were aged 25-35 years (90 (44.1%)), followed by 36-45 years (67 (32.8%)) and 46-55 years (47 (23.1%)). There was a slight male predominance (114 (55.8%)), with females constituting 90 (44.2%) of the sample. With respect to clinical experience, 57 (27.9%) had less than five years of experience, 34 (16.7%) had five-10 years, 49 (24.0%) had 11 to 15 years, and 64 (31.4%) had more than 15 years of experience. The majority of respondents were employed in academic institutions (159 (77.9%)), while 45 (22.2%) worked in private practice (Table 1).

**Table 1:** Demographic Characteristics and Clinical Practice Patterns (n = 204)

Variable	Category	Frequency (n)	Percentage (%)
Age	25-35 years	90	44.1%
	36-45 years	67	32.8%
	46-55 years	47	23.1%
Gender	Female	90	44.2%
	Male	114	55.8%
Years of Experience	<5 years	57	27.9%
	5-10 years	34	16.7%
	11-15 years	49	24.0%
	>15 years	64	31.4%
Practice Setting	Private practice	45	22.2%
	Academic institution	159	77.9%
Provide Interceptive Orthodontics	Yes	159	77.9%
	Considering it	34	16.7%
	No	11	5.4%
Recommendation Frequency	Frequently	125	61.2%
	Occasionally	68	33.3%
	Rarely	11	5.5%

Clinical practice patterns in interceptive orthodontics

Most pediatric dentists (159 (77.9%)) reported that they routinely provide interceptive orthodontic treatment in their practice, while 34 (16.7%) were considering incorporating it, and 11 (5.4%) stated that they currently do not provide interceptive orthodontics (Table 1). When eligible cases were encountered, 125 (61.2%) indicated that they frequently recommended interceptive orthodontic treatment, 68 (33.3%) did so occasionally, and only 11 (5.5%) reported recommending it rarely.

Common interceptive modalities and indications

**Figure 1:**
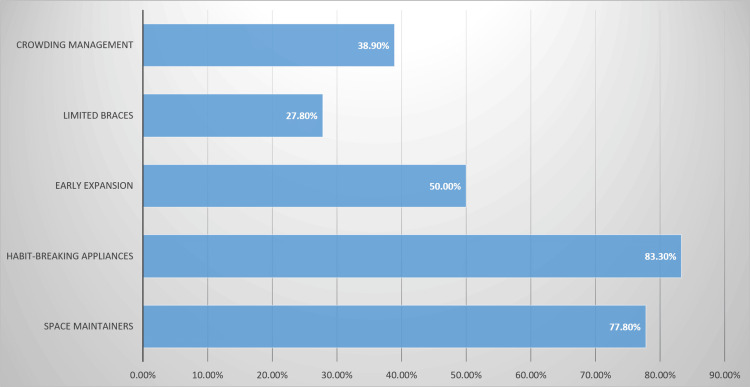
Recommended Interceptive Treatments

The most frequently recommended interceptive modalities (multiple responses allowed) are shown in Figure 1.

Habit-breaking appliances were the most commonly recommended option (159 (78.0%)), followed by space maintainers (148 (72.5%)). Early expansion was recommended by 100 (49.0%) respondents, crowding-management strategies by 79 (38.7%), and limited braces by 58 (28.4%).

**Figure 2:**
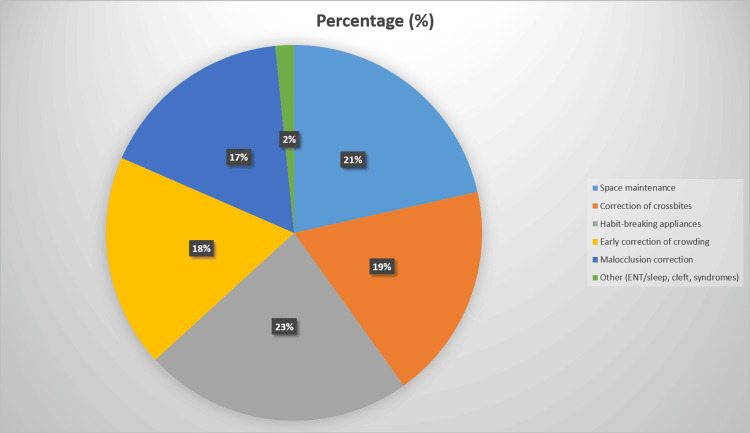
Indications for Interceptive Orthodontics

The main indications for initiating interceptive orthodontics are summarized in Figure 2.

The most common indication was habit-related problems (e.g., digit sucking, tongue thrust), reported by 159 (78.0%) respondents, followed closely by space maintenance after premature loss of primary teeth (148 (72.5%)). Correction of crossbites was cited by 128 (62.7%), early correction of crowding by 126 (61.8%), and broader malocclusion correction by 116 (56.9%). A smaller proportion (11 (5.4%)) reported using interceptive orthodontics in the context of ENT/sleep-related problems, cleft conditions, or syndromic cases.

Perceptions and beliefs regarding interceptive orthodontics

Exactly 102 (50.0%) respondents strongly agreed, and 79 (38.7%) agreed that interceptive treatment is effective in reducing the complexity of subsequent comprehensive orthodontic treatment, while 23 (11.3%) were neutral. With respect to cost-effectiveness, 102 (50.0%) believed that interceptive orthodontics is cost-effective for families, 90 (44.2%) felt that cost-effectiveness depends on the case, and 12 (5.8%) considered it not cost-effective. Perceptions of treatment duration were more varied: 90 (44.2%) believed that interceptive orthodontic protocols are longer than delayed comprehensive orthodontic treatment, whereas 57 (27.9%) felt the duration is about the same, and 57 (27.9%) perceived interceptive treatment to be shorter than initiating treatment later (Table 2).

**Table 2:** Perceptions, Challenges, and Future Intent (n = 204) *Percentages calculated from multiple responses.

Variable	Category	Frequency (n)	Percentage (%)
Effectiveness in Reducing Complex Treatment	Strongly agree	102	50.0%
	Agree	79	38.7%
	Neutral	23	11.3%
Satisfaction With Outcomes	Very satisfied	102	50.0%
	Moderately satisfied	90	44.2%
	Neutral	12	5.8%
Continue as First-Line Treatment	Very likely	136	66.7%
	Moderately likely	34	16.7%
	Neutral	34	16.7%
Main Challenges	Lack of parental understanding	128	62.7%*
	Compliance issues	118	57.8%*
	Difficulty managing children	116	56.9%*
	High treatment cost	91	44.6%*

Challenges in implementation

Respondents reported several perceived barriers to implementing interceptive orthodontics in routine pediatric dental practice. The most frequently cited challenge was lack of parental understanding or awareness, reported by 128 (62.7%) pediatric dentists. Compliance issues (e.g., poor appliance wear) were mentioned by 118 (57.8%), and difficulty managing cooperation with children by 116 (56.9%). High treatment cost was considered a barrier by 91 (44.6%), and inadequate curriculum and training during postgraduate education was noted by 12 (5.9%).

Analysis of the open-ended responses revealed several recurring themes. The most common was a call for better training and education in interceptive orthodontics (65 (31.9%)), including a stronger emphasis in the postgraduate curriculum and more structured exposure. Other frequently mentioned themes included the need for improved appliance compliance (45 (22.1%)), increased awareness among parents and the public (43 (21.1%)), greater access to workshops and hands-on courses (35 (17.2%)), and technical improvements in appliance design or integration with clear aligners (16 (7.8%)).

Satisfaction and future intent

**Figure 3:**
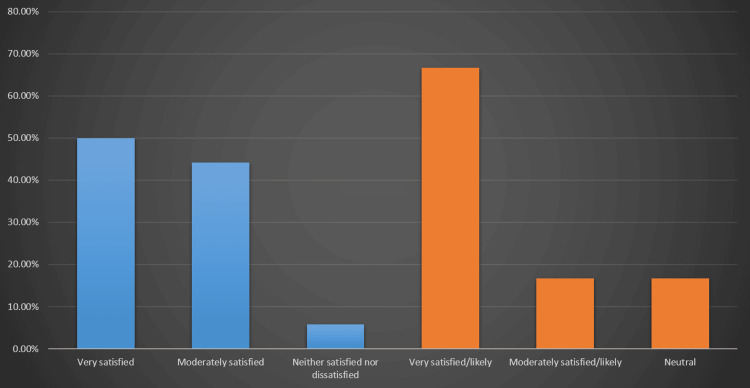
Satisfaction With Interceptive Orthodontic Outcomes and Intention to Continue Offering Interceptive Orthodontics as a First-Line Treatment Among Pediatric Dentists Blue bars represent levels of satisfaction with treatment outcomes; orange bars represent the likelihood of continuing to offer interceptive orthodontics as a first-line treatment.

Most pediatric dentists reported being satisfied with their clinical outcomes from interceptive orthodontic treatment (Figure 3).

Half of the respondents (102 (50.0%)) were very satisfied, 90 (44.2%) were moderately satisfied, and only 12 (5.8%) described themselves as neither satisfied nor dissatisfied. Regarding future intent, approximately two-thirds (136 (66.7%)) indicated that they were very likely to continue offering interceptive orthodontics as a first-line treatment where appropriate, 34 (16.7%) were moderately likely, and 34 (16.7%) were neutral about continuing to use interceptive orthodontics as a primary option.

Associations between respondent characteristics and provision of interceptive orthodontics

Chi-square analysis was performed to explore the association between key respondent characteristics and the routine provision of interceptive orthodontics (Table 3).

**Table 3:** Association Between Respondent Characteristics and Routine Provision of Interceptive Orthodontics (χ² test, N = 204) *Statistically significant at p < 0.05. Totals: Provides interceptive orthodontics = 159, considering/no = 45, overall N = 204.

Variable	Category	Provides interceptive orthodontics n (%)	Considering/No n (%)	χ² (df)	p-value
Age (Years)	25-35	76 (84.4)	14 (15.6)	7.71 (2)	0.021*
	36-45	53 (79.1)	14 (20.9)	-	-
	46-55	30 (63.8)	17 (36.2)	-	-
Years of Experience	<5	38 (66.7)	19 (33.3)	9.28 (3)	0.026*
	5-10	25 (73.5)	9 (26.5)	-	-
	11-15	39 (79.6)	10 (20.4)	-	-
	>15	57 (89.1)	7 (10.9)	-	-
Practice Setting	Private practice	30 (66.7)	15 (33.3)	3.47 (1)	0.063
	Academic	129 (81.1)	30 (18.9)	-	-
Gender	Female	76 (84.4)	14 (15.6)	3.31 (1)	0.069
	Male	83 (72.8)	31 (27.2)	-	-

A statistically significant association was observed between age and providing interceptive orthodontic treatment (χ² = 7.71, df = 2, p = 0.021). Pediatric dentists aged 25-35 years and 36-45 years were more likely to report routinely providing interceptive orthodontics (84.4% and 79.1%, respectively) compared with those aged 46-55 years (63.8%). Similarly, years of clinical experience showed a significant association with provision of interceptive care (χ² = 9.28, df = 3, p = 0.026). Among respondents with more than 15 years of experience, 89.1% reported routinely providing interceptive orthodontics, compared with 79.6% of those with 11-15 years, 73.5% with five-10 years, and 66.7% with less than five years of experience. The practice setting showed a non-significant trend towards higher provision of interceptive orthodontics among academic dentists compared with those in private practice (81.1% vs. 66.7%; p = 0.063), and gender showed a non-significant trend towards higher provision among female than male pediatric dentists (84.4% vs. 72.8%; p = 0.069).

## Discussion

Interceptive orthodontics, delivered in the primary and mixed dentitions, aims to interrupt the progression of developing malocclusions, improve arch development, and reduce the need for extraction‐based or surgical treatment in adolescence [10]. Early intervention can modulate craniofacial growth and dentoalveolar adaptation when sutural, periosteal, and condylar growth potential is still high, and when habits and functional disturbances can be corrected [11]. Clinical and epidemiological data indicate that a substantial proportion of children below 10 years of age present with conditions such as crossbite, crowding, and increased overjet that warrant preventive or interceptive measures, with higher prevalence in the eight- to 10-year age band [12]. At the same time, contemporary pediatric dentists are increasingly expected to provide basic orthodontic services-space maintenance, habit interception, management of anterior crossbite, and simple arch development-rather than limiting their role to referral [13]. However, most existing surveys have focused either on general practitioners and non-orthodontic specialists or on clinical prevalence of interceptive need, with limited data on how pediatric dentists in India perceive and integrate interceptive orthodontics into routine practice [14]. Against this background, our survey was designed to quantify Indian pediatric dentists’ perceptions, self-reported practices, and perceived barriers related to interceptive orthodontics.

Overall, our findings of broadly positive attitudes toward early diagnosis and management of malocclusion, and frequent use of space maintainers, habit-breaking appliances, and simple anterior crossbite correction, are consistent with contemporary literature on pediatric dentists’ scope of practice. Al-Dulaimy et al. reported that pedodontists from multiple countries felt reasonably confident in diagnosing malocclusions and most commonly provided space maintainers, habit breakers, anterior crossbite management, and serial extraction in their clinics, while simultaneously expressing strong interest in further education on preventive and interceptive orthodontics [13]. Decusară et al. illustrated, through a series of clinical cases, that functional and removable appliances used in the early mixed dentition can stimulate alveolar development, gain space, and reduce the severity of dental crowding, thereby simplifying later fixed-appliance therapy and often avoiding extractions [11]. Similarly, Rahman et al. showed that although the recorded prevalence of preventive and interceptive need in children under 10 years was relatively low (6.03%), crossbite and crowding were the most common problems, and children aged eight to 10 years had a higher burden of malocclusion than younger children, underscoring the window of opportunity that many of our respondents also recognized [12]. Together, these studies support our observation that pediatric dentists are aware of the importance of early orthodontic intervention and are already providing a meaningful range of interceptive services in the mixed dentition.

At the same time, some of our observations contrast with reports from other contexts, highlighting persistent gaps and variability. While our respondents reported a generally favorable perception of interceptive orthodontics and regular provision of basic procedures, surveys among general dentists and non-orthodontic specialists have demonstrated more modest knowledge levels and inconsistent diagnostic and referral practices [15]. Alshami et al. found that the appropriate age to start orthodontic treatment was poorly understood by many practitioners, and that a substantial proportion did not routinely carry out orthodontic diagnostic procedures, despite acknowledging the impact of habits and malocclusion on function and aesthetics [14]. Furthermore, case-based evidence suggests that early functional and orthopedic approaches, including arch expansion and growth-modifying appliances, can substantially modify arch form and skeletal relationships [16], yet our respondents, similar to those in Al-Dulaimy et al., appeared more comfortable with simpler procedures than with comprehensive orthopedic correction [13]. Finally, Berk et al. demonstrated high agreement among orthodontists, pediatric dentists, and general practitioners in judging orthodontic treatment need [15], whereas our data suggest that, in everyday pediatric practice, translation of this diagnostic agreement into active interceptive care may still be influenced by factors such as practice setting, training exposure, and access to specialist support.

This study has several limitations that should be acknowledged alongside its strengths. First, although the a priori sample size calculation indicated a minimum of 267 participants, only 204 complete responses were obtained. This represents a small proportion of the estimated pediatric dentist workforce in India, reduces the precision of the estimates, and limits the generalizability of the findings and robustness of subgroup analyses. Second, the cross-sectional, questionnaire-based design relies on self-reported perceptions at a single time point and is susceptible to recall and social-desirability bias. In addition, dissemination through professional networks, institutional mailing lists, and social media constituted a non-probability, voluntary-response sampling strategy, which may have over-represented dentists affiliated with academic institutions and under-represented those in remote or resource-limited settings. Finally, we did not collect detailed information on respondents’ postgraduate training in interceptive orthodontics, including where they were trained (country/program) and the extent or level of competency to which interceptive orthodontics is taught. As a result, we could not account for heterogeneity in training background when interpreting differences in perceptions, confidence, and reported practices. Future research using larger, probability-based samples and incorporating detailed training and curriculum-related variables is needed to address these limitations. Conversely, key strengths include ethics approval from a recognized university ethics board, a relatively diverse national sample spanning multiple regions and practice settings, and a focus specifically on pediatric dentists rather than mixed-specialty groups. By combining items on knowledge, attitudes, reported practices, and perceived barriers, the survey provides a nuanced snapshot of where interceptive orthodontics currently sits within Indian pediatric dentistry.

Beyond patient- and system-level factors, training itself is likely a key barrier. In many Indian pediatric dentistry programs, exposure to interceptive orthodontics during residency may be limited, and structured continuing-education opportunities in this area are variably available within India and abroad. Such gaps in initial and ongoing training could contribute to the variability in confidence, perceived scope, and utilization of interceptive procedures observed in this survey, and should be a priority target for curriculum development and CE planning.

Future research should move beyond cross-sectional self-report to prospective and interventional designs that can evaluate how targeted continuing-education programs, structured postgraduate curricula, and collaborative care pathways with orthodontists influence actual interceptive case-load and child-level outcomes. Multi-center clinical audits and registry-based studies could corroborate self-reported practices with treatment records, while qualitative work with pediatric dentists and parents may clarify contextual barriers such as chair-time, remuneration, parental preferences, and perceived complexity of appliances. Comparative surveys, including general dentists and orthodontists, would help map referral networks and identify missed opportunities for early interception. Finally, integrating interceptive orthodontics with emerging modalities, such as digital diagnostics and risk-based screening, offers a promising avenue for research and service development.

## Conclusions

Within this nationwide sample of pediatric dentists in India, the survey identified distinct patterns in awareness, reported diagnostic practices, use of interceptive procedures, and referral behaviors related to developing malocclusions. These patterns, as reflected in the descriptive and comparative analyses presented, suggest that while pediatric dentists recognize the importance of interceptive orthodontics, there remain gaps and variability in how such care is discussed, initiated, and coordinated in clinical practice. Taken together, the findings highlight opportunities to strengthen early identification and management of malocclusions through targeted continuing education, clearer referral pathways, and closer collaboration with orthodontists across the pediatric age range.

However, these conclusions must be interpreted in light of the study’s methodological limitations, including its cross-sectional, self-reported design, modest sample size drawn through non-probability online dissemination, and the absence of detailed information on respondents’ postgraduate training and local service contexts. These factors may limit generalizability and do not allow inferences about causality or the direction of any observed associations. Future research using larger, more representative samples, objective clinical data, and designs capable of addressing potential confounders is needed to confirm and extend these observations.

## Appendices

Survey on practices of pediatric dentists regarding interceptive orthodontics

Demographic Information

1. Age

o   25-35 years

o   36-45 years

o   46-55 years

o   56 years and above

2. Gender

o   Male

o   Female

o   Prefer not to say

3. Years of experience in Pediatric Dentistry

o   Less than five years

o   Five to 10 years

o   11-15 years

o   More than 15 years

4. Practice setting

o   Private practice

o   Academic institution

o   Government institution

o   Other (please specify):

Perceptions on Interceptive Orthodontics

5. Do you routinely provide interceptive orthodontic treatments to pediatric patients?

o   Yes

o   No

o   Considering it

6. How often do you recommend interceptive orthodontic treatments?

o   Frequently

o   Occasionally

o   Rarely

o   Never

7. What are the main indications for interceptive orthodontics in your practice? (Select all that apply)

o   Correction of crossbites

o   Habit-breaking appliances

o   Early correction of crowding

o   Malocclusion correction

o   Other (please specify):

8. Do you believe interceptive orthodontics effectively reduces the need for complex orthodontic treatments later?

o   Strongly agree

o   Agree

o   Neutral

o   Disagree

o   Strongly disagree

9. Which interceptive treatments do you most frequently recommend? (Select all that apply)

o   Habit-breaking appliances

o   Limited braces

o   Early expansion

o   Other (please specify):

Challenges With Interceptive Orthodontics

10. What are the main challenges you face in implementing interceptive orthodontics? (Select all that apply)

o   Lack of parental understanding

o   Compliance issues

o   High treatment cost

o   Difficulty in managing cooperation with young children

o   Other (Please specify):

11. Do you find that interceptive orthodontics adds significant treatment duration compared to delayed orthodontics?

o   Yes

o   No

o   About the same

12. Are interceptive treatments cost-effective for patients compared to delayed orthodontic treatments?

o   Yes

o   No

o   Depends on the case

General Opinions

13. In the past 12 months, how satisfied are you with the outcomes of interceptive orthodontic treatments in your practice?

o   Very satisfied

o   Moderately satisfied

o   Neutral

o   Unsatisfied

14. Would you continue to offer interceptive orthodontics as a first-line treatment for pediatric patients?

o   Definitely

o   Probably

o   Unlikely

o   Not at all

15. What improvements would you like to see in the field of interceptive orthodontics? (Open-ended)

16. Additional comments or suggestions regarding interceptive orthodontic treatments: (Open-ended)
